# Evaluating the impact of mobility in COVID-19 incidence and mortality: A case study from four states of Mexico

**DOI:** 10.3389/fpubh.2022.877800

**Published:** 2022-08-04

**Authors:** César Arturo Méndez-Lizárraga, MLucía Castañeda-Cediel, Guadalupe Delgado-Sánchez, Edith Elizabeth Ferreira-Guerrero, Leticia Ferreyra-Reyes, Sergio Canizales-Quintero, Norma Mongua-Rodríguez, Norma Tellez-Vázquez, María Eugenia Jiménez-Corona, Kathryn Bradford Vosburg, Omar Y. Bello-Chavolla, Lourdes García-García

**Affiliations:** ^1^Centro de Investigación Sobre Enfermedades Infecciosas, Instituto Nacional de Salud Pública, Cuernavaca, Mexico; ^2^Posgrado en Geografía, Facultad de Filosofía y Letras, Universidad Nacional Autónoma de México, Mexico City, Mexico; ^3^Departamento de Epidemiología, Instituto Nacional de Cardiología “Ignacio Chávez”, Mexico City, Mexico; ^4^Institute for Global Health Sciences, University of California, San Francisco, San Francisco, CA, United States; ^5^Dirección de Investigación, Instituto Nacional de Geriatría, Mexico City, Mexico

**Keywords:** mobility, COVID-19, incidence, correlation, Mexico, change-point, mortality

## Abstract

**Introduction:**

The COVID-19 pandemic in Mexico began at the end of February 2020. An essential component of control strategies was to reduce mobility. We aimed to evaluate the impact of mobility on COVID- incidence and mortality rates during the initial months of the pandemic in selected states.

**Methods:**

COVID-19 incidence data were obtained from the Open Data Epidemiology Resource provided by the Mexican government. Mobility data was obtained from the Observatory for COVID-19 in the Americas of the University of Miami. We selected four states according to their compliance with non-pharmaceutical interventions and mobility index. We constructed time series and analyzed change-points for mobility, incidence, and mortality rates. We correlated mobility with incidence and mortality rates for each time interval. Using mixed-effects Poisson models, we evaluated the impact of reductions in mobility on incidence and mortality rates, adjusting all models for medical services and the percentage of the population living in poverty.

**Results:**

After the initial decline in mobility experienced in early April, a sustained increase in mobility followed during the rest of the country-wide suspension of non-essential activities and the return to other activities throughout mid-April and May. We identified that a 1% increase in mobility yielded a 5.2 and a 2.9% increase in the risk of COVID-19 incidence and mortality, respectively. Mobility was estimated to contribute 8.5 and 3.8% to the variability in incidence and mortality, respectively. In fully adjusted models, the contribution of mobility to positive COVID-19 incidence and mortality was sustained. When assessing the impact of mobility in each state compared to the state of Baja California, increased mobility conferred an increased risk of incident positive COVID-19 cases in Mexico City, Jalisco, and Nuevo León. However, for COVID-19 mortality, a differential impact of mobility was only observed with Jalisco and Nuevo León compared to Baja California.

**Conclusion:**

Mobility had heterogeneous impacts on COVID-19 rates in different regions of Mexico, indicating that sociodemographic characteristics and regional-level pandemic dynamics modified the impact of reductions in mobility during the COVID-19 pandemic. The implementation of non-pharmaceutical interventions should be regionalized based on local epidemiology for timely response against future pandemics.

## Introduction

During the first months of the SARS-CoV-2 pandemic, Mexico concentrated 8.6% of confirmed cases across the Americas by July 2020, a region that represented 25% of the world cases (238,511 cases in Mexico and 2,746,277 in Latin America) (1). The first three cases of SARS-CoV-2 in the country were confirmed on February 28th, 2020 (2), a month after the World Health Organization (WHO) declaration of the epidemic as a Public Health Emergency of International Concern (3). As the world faced this novel pathogen, no specific therapeutics and vaccines were available, forcing the global community to appeal to nonpharmaceutical interventions (NPIs) (4, 5).

The Mexican government officially published public health mitigation strategies by late March (March 24th, 2020) (6); days later, the Consejo de Salubridad General (General Health Council, GHC) formally recognized the pandemic as a national health emergency (7). This occurred when more than 1,000 cases and 28 deaths of COVID-19 had been confirmed, and ongoing local transmission took place in the country (8, 9). A combination of NPIs, titled Jornada Nacional de Sana Distancia (National Program of Safe Distance, NPSD), centered around the suspension of non-essential activities to slow viral transmission, hospitalizations, and fatalities, was carried out starting March 30th, 2020 (10) resulted in a decline in population mobility during the following weeks in all 32 states. Other interventions included the promotion of physical distancing and handwashing during the early stages (11, 12). As outlined by WHO's guidance, a critical component of national and regional response to a pandemic is timely and effective interventions, such as restricting the movement of people and goods, which allow for time to implement preparedness activities and slow viral transmission (4).

The NPSD concluded on May 30th as the authority concerning health policies for SARS-CoV-2 mitigation, and guidance for the return to non-essential activities (and thus population mobility) was transferred from the federal government to state governments for the new normality (11, 12). National Health authorities developed an epidemiological risk tool: semáforo epidemiológico (epidemiological traffic light), based mainly on transmission, hospitalizations, fatalities, and hospital bed availability to guide state decision-makers (13). NPIs continued to be encouraged by all health authorities throughout the new normality (14).

The country's initial response to COVID-19 presents an opportunity to assess the impact of NPIs on mitigating health damages from the pandemic and provides a unique opportunity for future pandemic preparedness and readiness vs. potential emerging and re-emerging pathogens. As outlined further in the present work, multiple studies have explored the relationship between population mobility and the trajectory of the pandemic, the first being very variable among reports; daily mobility (measured by Google Maps and Apple), average mobility across time segments, mobility characterized by place of occurrence (example: house, supermarket, public transport, recreational spaces), internal mobility within a city, mobility between regions or states and international mobility (15–26). In this study, we studied four states (Baja California, Nuevo León, Jalisco, and Mexico City) based on mobility, compliance to NPIs, and metropolitan area. We conducted a three-step analysis in four states to evaluate the impact of mobility on COVID-19 incidence and mortality using (1) a change point analysis, (2) correlation analysis to examine the relationship between mobility and incidence and mortality rates, and (3) adjusted mixed-effects Poisson models, for evaluation of the impact of reductions in mobility on the incidence rate of positive COVID-19 cases and COVID-19 deaths.

## Materials and methods

### Setting

**Figure 1:**
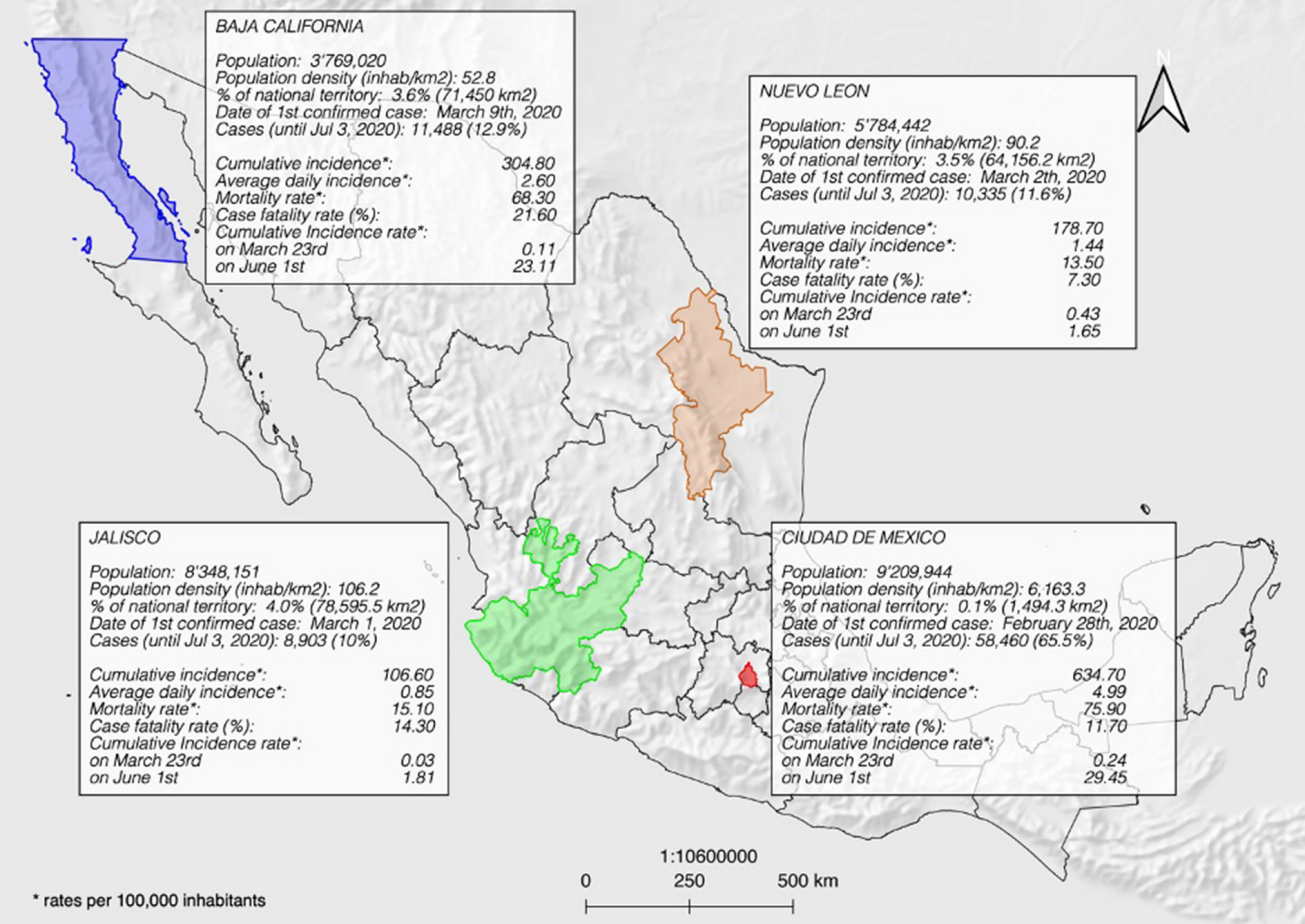
The geographical location of the states studied and general characteristics of the COVID-19 pandemic, Mexico. The map shows the population of each state, its density per km^2^, the percentage it represents from the country's territory, the date of the first confirmed cases, and confirmed cases until July 3, 2020. It also shows cumulative incidence, average daily incidence, the mortality rate per 100,000 habitants, case fatality rate, and the cumulative incidence rate on March 23rd, 2020 (beginning of the National Program of Safe Distance) and June 1st, 2020 (beginning of the New Normality).

Mexico's territory includes 1,960,189 km2 (10.2% of Latin America's extension) and has 126,014,024 inhabitants, with a population density of 64.28 inhabitants/km2 (27). Out of thirty-two states, four were selected for this study: Baja California, Nuevo León, Jalisco, and Mexico City; all comprise five of the top fifteen metropolitan areas in the country (28). Figure 1 shows their location and relevant data.

The Ministry of Health coordinates the health system, which is fragmented into several subsystems that organize, provide, and regulate most of its services. The three main components operate in parallel and include: (a) multiple employment-based social insurance schemes, (b) public assistance services for the uninsured, and (c) a private sector composed of service providers and insurers (29).

### Data and sample selection

Open data on COVID-19 cases in Mexico were drawn from the General Directorate of Epidemiology repository for incidence and mortality rates (30). In the case of mobility, data was extracted from the Observatory for COVID-19 in the Americas of the University of Miami (adapted from the Oxford Government Response Tracker, OxCGRT 5.0, and from Google population mobility) for Mexico and its thirty-two states (31). Google provided mobility measurements on travel to workplaces, supermarkets and pharmacies, parks and plazas, public transportation stations, shops, and places for recreation, excluding mobility from the residential category. The mobility index reflects the seven-day moving average for mobility data on visits to the mentioned sites instead of a daily value. This average is a more stable indicator and reflects the overall trend regardless of small daily fluctuations (31). We used a daily public policy index (PPI) that measures compliance to non-pharmaceutical measures (31) and date of implementation (graded on a 0–100 scale), and population mobility (reported as a change in percentage based on early 2020 mobility). All states were drawn on a Cartesian plane and categorized into four groups based on the median in mobility and PPI index (low mobility and low PPI, low mobility and high PPI, high mobility and high PPI, high mobility, and low PPI) (31). The state with the largest metropolitan area from each group was selected, representing different adherence to non-pharmaceutical interventions as described above. Because restrictions on population mobility were implemented during the first weeks of the pandemic, the present study aims to exhibit the impact of these restrictions in the early stages of viral transmission (the first confirmed case in Mexico) up to the first week after restrictions were eased (July 3rd, 2020). An exploratory analysis was made for each state's epidemiology. Analyzed data is available in an external repository (32).

### Change-point analysis

Time series for the independent variable (mobility) and dependent variables (incidence and mortality) were constructed. A change-point analysis utilized the R package *ecp* (33) for the three described variables from each state in RStudio. The ecp package performs a retrospective analysis of an entire sequence that estimates both the number of change points and the places in time in which they occur. It can perform time series on either univariate or multivariate parameters without a priori knowledge of the number of change points, working without any assumptions about the nature of change or any distribution hypothesis beyond the existence of the absolute moment, for some (0, 2)^*^. Estimation is based on hierarchical clustering.

We used a divisive algorithm (*e-divisive*) that has shown consistent estimates of the number and location of change points. Divisive estimation sequentially identifies change points *via* a bisection algorithm and can detect any distributional change within the data. The multiple change points are estimated by iteratively applying a procedure for locating a single change point. A new transition point location is calculated to divide an existing segment at each iteration. As a result, the progression of this method can be diagrammed as a binary tree.

Additionally, the statistical significance of an estimated change point is determined through a permutation test. Specifications for running the analysis included the number of iterations (199) and the level of statistical significance (set at ≤0.05). The time complexity of this method is ϑ(kT2), where k is the number of estimated change points, and T is the number of observations in the series (34).

The ecp package was selected due to a better fit of the data from the sample, although there are other packages for change-point analysis (35).

### Estimating the effects of mobility on incidence and mortality

We created time intervals based on incidence and mortality change-points. For incidence rates, the interval was constructed taking 12 days before its change-point, considering the mean incubation period for SARS-CoV-2 and delays in seeking medical attention. Then, the endpoint of each segment was fixed 14 days after the change-point in incidence. Finally, we used the same approach for mortality but with a difference of 28 days (36, 37). Afterward, we estimated the impact of mobility on both incidence and mortality by calculating Spearman's rank-order correlation coefficient for each segment (38). As observed in the Supplementary materials, intervals include both before and after tendencies based on identified change-points for all analyzed variables and Spearman's correlation coefficient.

To evaluate the impact of reductions in mobility on the incidence rate of positive COVID-19 cases and COVID-19 deaths in all states being assessed, we fitted a mixed-effects Poisson regression model fit by maximum likelihood using the Laplace Approximation, which took the state of origin and date of symptom onset as random intercepts to account for the dependence of COVID-19 rates across time and regional-level pandemic dynamics, incorporated log-transformed population as the regression offset. Next, we obtained incidence rate ratios (IRR) by exponentiating the beta coefficients obtained from the mixed-effects models. All models were adjusted for the number of physicians and available hospital beds per 10,000 inhabitants as a proxy of the impact of the availability and access to medical services in the evaluated states and the percentage of the population living in poverty (39). To assess the impact of mobility in each state, we fitted a mixed-effects Poisson model with a random effect in the date of symptom onset and an interaction effect with the state of case identification for both COVID-19 incidence and mortality. All models were evaluated using residual diagnostics, evaluation of overdispersion, and assessment of the influence of random effects in the model. Models were selected by minimization of the Bayesian Information Criterion.

This study was ruled “Exempt from Review” by the “Ethical Commission” of the Instituto Nacional de Salud Pública (approval number PT651) because the database is public.

## Results

**Figure 2:**
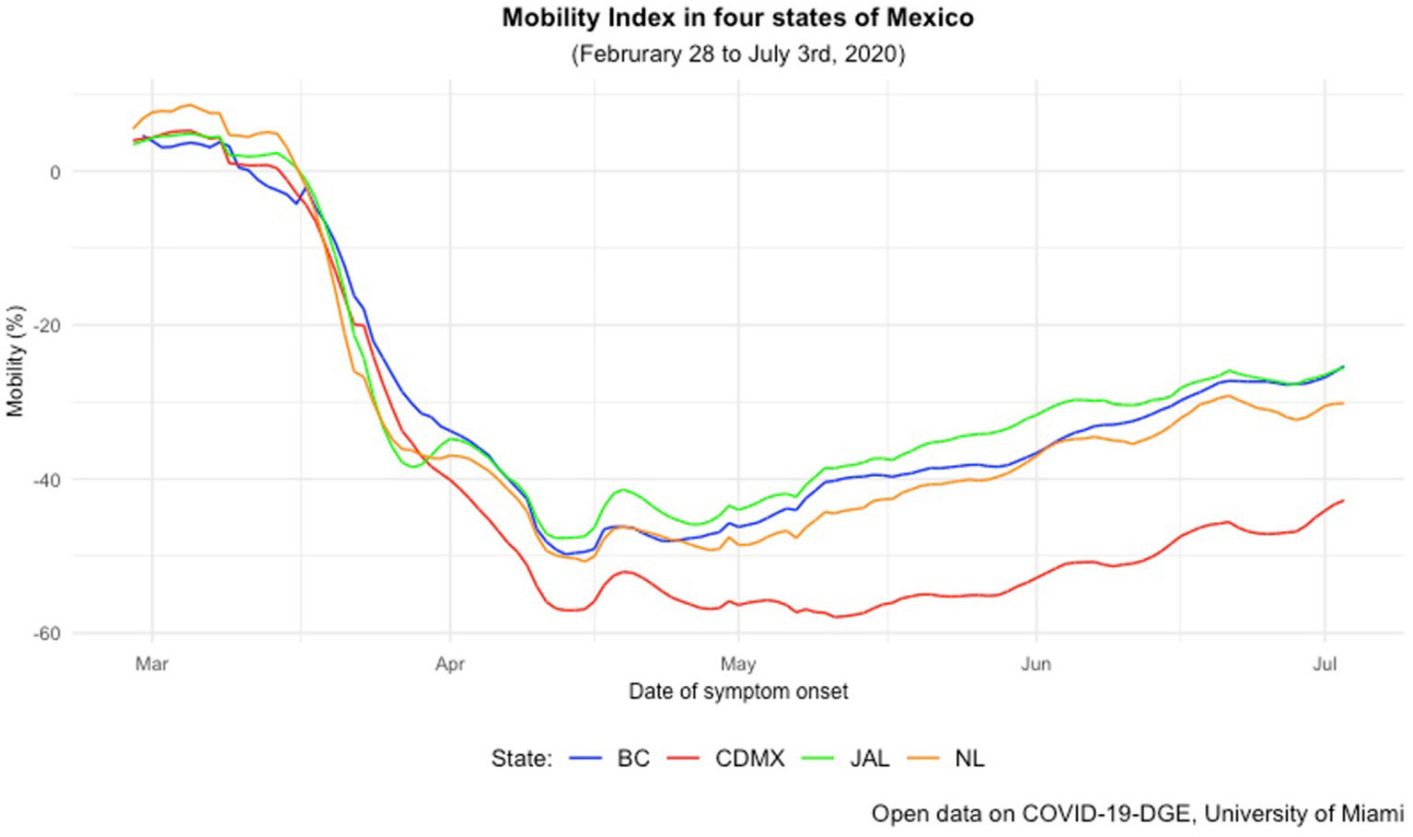
Mobility index in four states from February 28th to July 3rd Mexico, 2020. The horizontal axis shows the period since the confirmation of the first COVID-19 case in Mexico, from February 28th, 2021, to July 3rd 2021. The vertical axis shows the percentage change in the mobility index documented by Google in a 10 to−60% range. The blue line represents the mobility index across the specified period in Baja California, the red line Ciudad de Mexico, the green line Jalisco and the yellow line Nuevo Leon. Data were retrieved from the Observatory for COVID-19 in the Americas, University of Miami.

Before the NPSD, mobility decreased across the four states, reaching its lowest levels in late April (Figure 2). Mexico City reduced its mobility by close to 60%. It maintained the smallest percentages compared to the rest of the states, which reached their lowest levels between−40 and−50% in early April. Halfway throughout the month, there was an increase in mobility that coincided with the first ending date of the NPSD established by the GHC (6) and national holidays from early April in some states (Table 1). After reassessing the spread of SARS-CoV-2, federal health authorities extended the campaign until the end of May 2020 (40), but mobility continued to increase steadily during the month. The change-point analysis in mobility revealed shifts in all four states during holidays (Table 1; Supplementary Figures 1–8).

**Table 1:** Critical dates during the first months of the pandemic in Mexico (2020).

**Critical dates during the first months of the pandemic in Mexico (2020)**	
*February 28th*	First confirmed cases in Mexico.
*March 23rd*	NPSD begins^*^
*March 27th*	COVID-19 is recognized as a severe disease that requires extraordinary measures and needs to be prioritized all over the country^*^
*March 30th*	A national health emergency is declared due to the SARS-CoV-2 pandemic^*^
*April 5-11*	Holy week holidays
*April 19th*	First ending date of the NPSD^*^
*April 21st*	Extension of the NPSD (up to May 30th)
*May 1st*	National holiday: Worker's Day
*May 5th*	National holiday: Cinco de Mayo (Battle of Puebla)
*May 10th*	National holiday: Mother's Day
*May 30th*	Ending of the NPSD
*June 1st*	Beginning of the “New Normality,” the epidemiological light is introduced. All states are in red (non-essential activities continue to be suspended)
*June 15th*	The epidemiological light changes to orange in Nuevo Leon and Jalisco. Ciudad de México and Baja California remain in red.

* Determined by the General Health Council. Besides critical dates signaling the evolution of the COVID-19 pandemic and the response from the Mexican government to it, the table mentions key dates from Mexico's culture, traditions, and holidays.

It is worth mentioning that many holidays are celebrated in April and May, like Labor Day and Easter, the latter being one of many holidays associated with the Christian religion. On the other hand, national festivities such as Cinco de Mayo, which commemorates a victory over French invaders in 1862, and Mother's Day, take a considerable part in celebrations across the country with family and friends (41, 42).

Starting June 1st, a gradual return to non-essential activities was guided and regulated on a state level (11), based on the traffic-light epidemiological risk tool (13, 14). As the new normality began, all states were on a red light, meaning that no non-essential activities were to be reinitiated until mid-June when Nuevo Leon and Jalisco changed to an orange light (43). However, mobility rose gradually in all four states across the new normality. The change-point analysis (Supplementary Figures 1–8) revealed a mobility shift during the Holy Week holiday weekend (April 5–11) in all four states. Another change in mobility that coincided with culturally important dates happened during Mother's Day weekend except in Mexico City. As mobility increased gradually over the following weeks, multiple change-points were identified across May and June, even after the new normality (Supplementary Figures 1–8).

**Figure 3:**
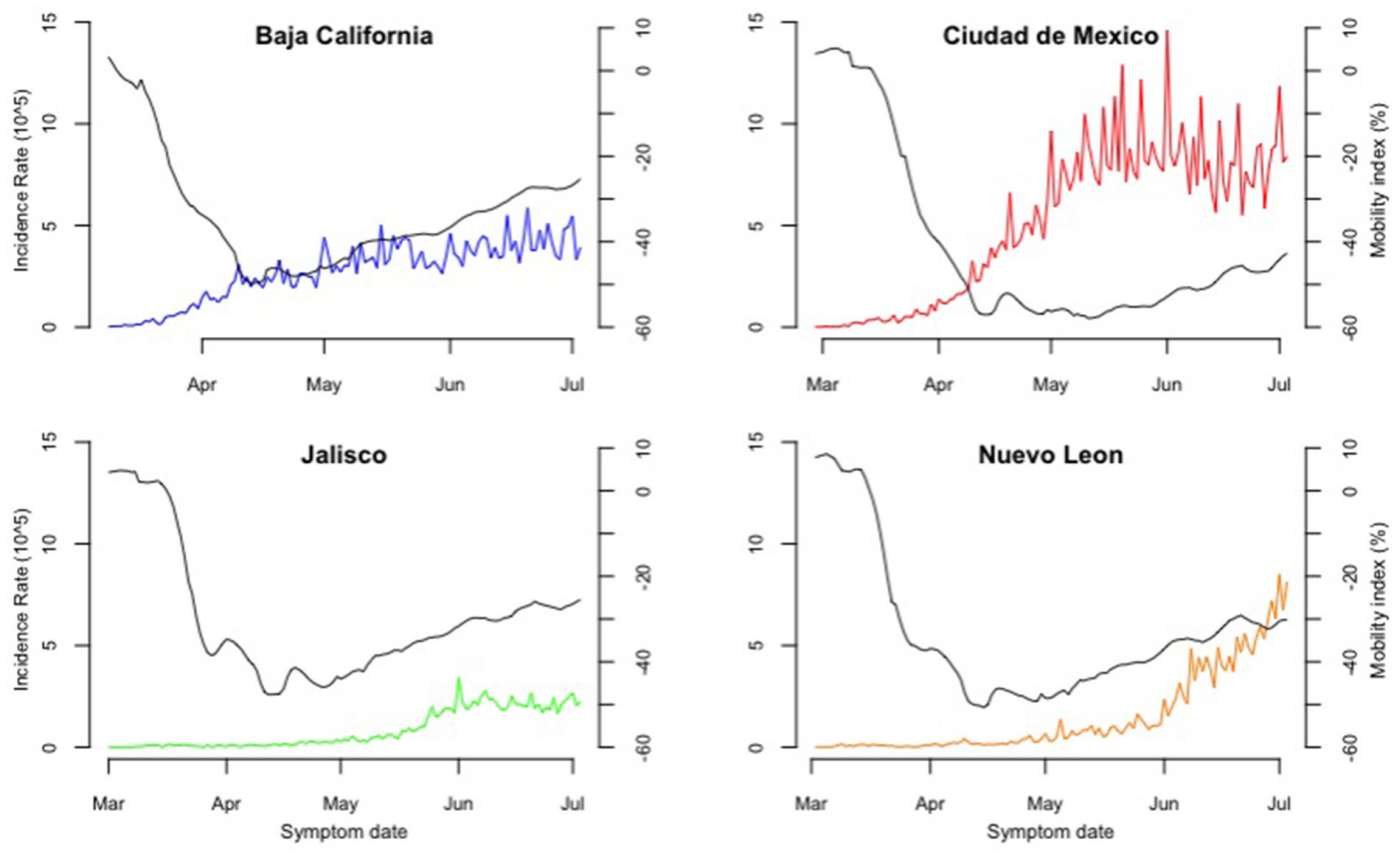
Incidence rate by symptom onset and mobility index in four states from February 28th to July 3rd, Mexico, 2020. The horizontal axis shows the period covered since the confirmation of the first COVID-19 case in Mexico, February 28th, 2021, to July 3rd 2021. The left vertical axis shows the incidence rate of confirmed cases per 100,000 habitants scale, and the right vertical axis shows the percentage change for mobility on a 10 to−60% range. The continuous black line represents the average daily mobility rate documented by Google. The colored line shows daily confirmed cases based on their date of symptom onset.

**Figure 4:**
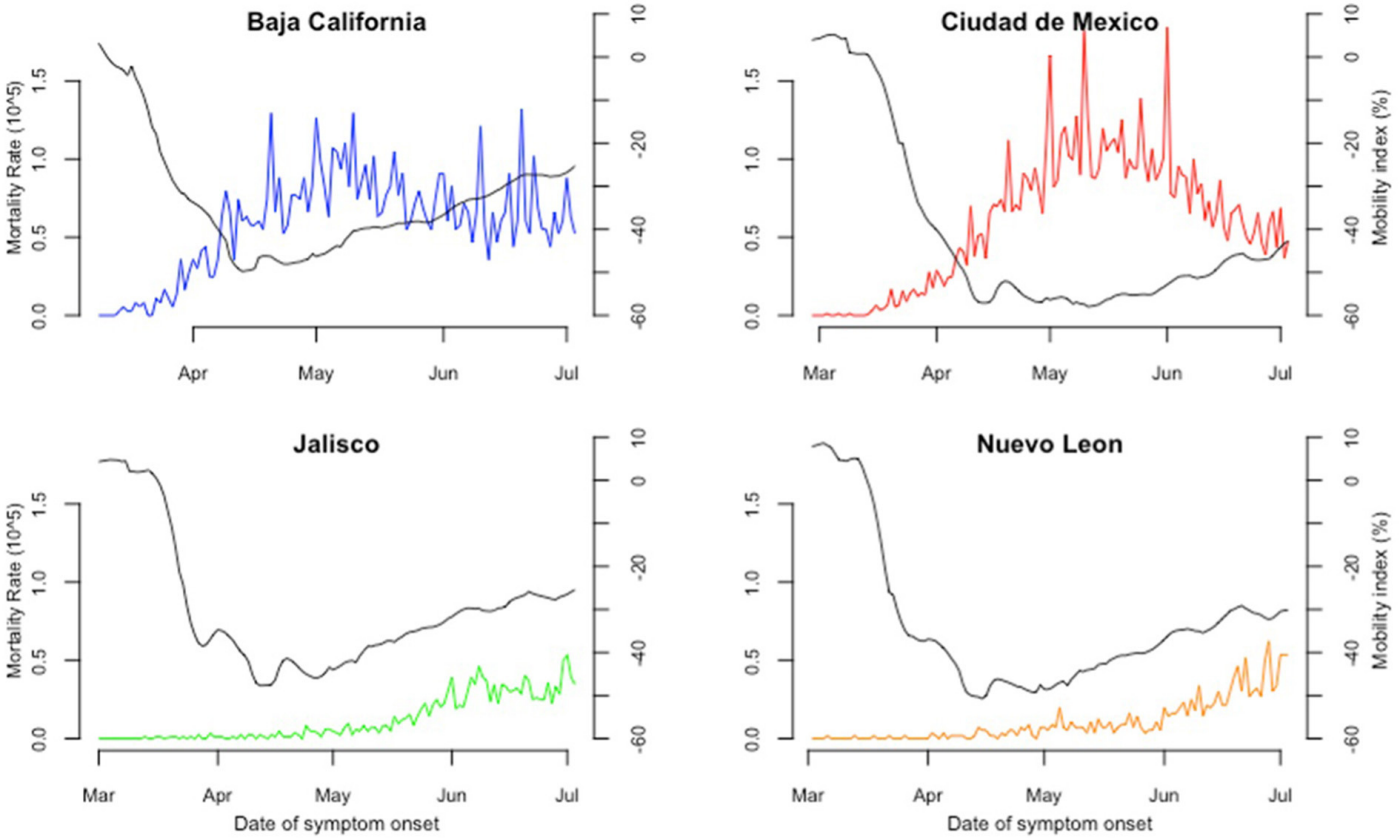
Mortality rate and mobility index in four states of Mexico from February 28th to July 3rd. The horizontal axis depicts the period covered since the confirmation of the first COVID-19 case in Mexico, February 28th, 2021, to July 3rd 2021. The left vertical axis shows the mortality rate, 0–1.5 confirmed COVID-19 deaths per 100,000 habitants, and the right vertical axis shows the percentage change for mobility in a 10 to−60% range. The continuous black line represents the average daily mobility rate documented by Google. The colored line shows daily confirmed cases based on their date of symptom onset.

During the first weeks after the first confirmed case, Baja California and Mexico City sustained an upward trend in their incidence rate well before mobility reached its lowest levels (Figure 3); both continued to increase during the following weeks after March 23rd. After experiencing similar incidence rates, Mexico City's escalated by mid-April, reaching its peak in June (14 cases per 100,000 habitants), while Baja California's increased on a lower scale across May and June (up to 6 cases per 100/000 habitants). Both states' mobility index increased gradually during the following months (Figure 2). Jalisco and Nuevo Leon had a lower incidence and mortality rates as mobility decreased (Figures 3, 4). Both states' incidence rates started to rise until mid-May, parallel to both mobility's increasing trends as seen in their respective Spearman's correlation coefficients (Table 2). Then, Nuevo Leon's incidence rate continued to escalate (up to 8 cases per 100,000 habitants), surpassing Baja California's and closing in on Mexico City's. At the same time, Jalisco maintained a steady and lower rate during the remaining period, regardless of the sustained rise in mobility (Figure 3).

**Table 2:** Spearman's rank correlation coefficients for mobility and incidence and mortality rates by time intervals from analyzed states in Mexico.

**State**		**Time segment (2020)**	**Incidence change point**	**Spearman coefficient (Incidence and mobility**		**Time segment (2020)**	**Mortality change point**	**Spearman coefficient (Mortality and mobility**
Baja California	1	March 17 to April 12	March 29	−0.86	1	March 11 to April 22	April 8	0.31
	2	March 27 to April 22	April 8	0.27	2	April 2 to May 14	April 30	−0.19
	3	April 8 to May 14	April 30	−0.05	3	April 25 to June 6	May 23	−0.19
	4	May 24 to June 19	June 5	−0.07				
Jalisco	1	April 8 to May 4	April 20	0.42	1	March 1 to April 13	March 30	−0.22
	2	April 18 to May 14	April 30	0.62	2	March 27 to May 8	April 24	0.21
	3	May 5 to 31	May 17	0.87	3	April 26 to June 7	May 24	0.5
	4	May 12 to June 7	May 24	0.68				
	5	May 20 to June 15	June 1	−0.09				
Nuevo León	1	April 22 to May 18	May 4	0.3	1	March 2 to April 15	April 1	−0.28
	2	May 23 to June 18	June 4	0.18	2	May 25 to 6	April 24	0.45
	3	June 13 to July 3	June 25	0.45	3	May 4 to June 15	June 1	0.41
Ciudad de México	1	March 25 to April 20	April 6	−0.35	1	February 28 to April 8	March 25	– 0.83
	2	April 8 to May 5	April 20	−0.18	2	March 18 to April 29	April 15	0.06
	3	April 19 to May 15	May 1	−0.23	3	April 8 to May 20	May 6	0.275
					4	May 14 to June 25	June 11	−0.13

Time segments were constructed based on mobility change-points; 12 days before change-points in incidence and 28 for mortality. Both periods for incidence and mortality ended after 14 days after their change-point date. Spearman's coefficients were calculated for both incidence and mobility and mortality and mobility in each state.

We identified change points in incidence 12–13 days after the original NPSD ending in Mexico City and Baja California. However, these were not preceded by changes in mobility near the dates. On the other hand, change-points in incidence in Jalisco and Nuevo León did come after change-points in mobility (8–16 days) (Supplementary Figures 1–4).

Both Baja California's and Mexico City's mortality rates had a similar trajectory. Rates rose even though mobility reached its lowest levels in both territories (Figure 4). Mexico City's reached its highest levels during May (1.7 deaths per 100,000 habitants), experimenting a steady decline during June, while Baja California's oscillated across the whole period on a lower scale (between 0.5–1.2 deaths per 100,000 habitants). During the first weeks, no increases in mortality rates were observed for both Jalisco and Nuevo León (Figure 4). Like both states' incidence rate trajectories, mortality rose as mobility and incidence surged throughout late May and June. Mortality rates in these two states attained similar values by the end of the study period (0.5 deaths per 100,000 habitants). By the end of the period analyzed, Baja California and Mexico City had at least four times the mortality rate compared to Nuevo Leon and Jalisco (68.3 and 75.9 vs. 15.1 and 13.5 deaths per 100,000 habitants). Regarding change-points, all four states had similar differences in days between change points in mobility and their respective mortality rate; differences ranged from 20 to 37 days and were present on most of the dates (Supplementary Figures 5–8).

Based on the construction of time intervals from change-points in incidence, positive correlations were obtained for incidence and mobility in Jalisco and Nuevo León, the first one having an overall higher magnitude. On the other hand, Baja California and Mexico City had mixed results for incidence and mobility, with both negative and positive correlations across their intervals, with no apparent pattern between both variables (Table 2; Supplementary Figures 9–12). Regarding mortality and mobility, correlations were also positive for Jalisco and Nuevo León, excluding the initial intervals where mobility experimented its initial decrease. As for Baja California and Mexico City, no pattern was identified as seen by mixed results in coefficients for mortality and mobility (Table 2; Supplementary Figures 13–16).

Overall, dependent variables (incidence and mortality rate) had an ascending change in their trajectories after their respective change-points, reinforcing the influence of mobility on our incidence and mortality hypothesis. This is not the case for Baja California and Ciudad de Mexico, as observed in their mobility and mortality trends (Supplementary Figures 13, 14), with changes in the opposite direction after their respective change-points and verified with Spearman's correlation coefficients.

When evaluating the mixed-effects Poisson model results, we identified that a 1% increase in mobility yielded a 5.2% increase in the risk of incident COVID-19 cases in all evaluated states. Mobility was estimated to contribute 8.5% to the variability in incident COVID-19 cases. For the case of COVID-19 mortality rates, we also identified a significant association between mobility and COVID-19 deaths, where a 1% increase in mobility was associated with a 2.9% increase in the risk of incident COVID-19 deaths. Nevertheless, the contribution of mobility was lower, representing 3.8% of the variability in COVID-19 mortality. In fully adjusted models, the contribution of mobility to positive COVID-19 incidence and COVID-19 mortality was sustained. Notably, in fully adjusted models, the percentage of the population living in poverty displayed a decreased risk for positive COVID-19 cases but an increased risk for COVID-19 mortality (Table 3). When assessing the impact of mobility in each state compared to the state of Baja California, we identified that increased mobility conferred an increased risk of incident positive COVID-19 cases in Mexico City, Jalisco, and Nuevo León. However, for COVID-19 mortality, a differential impact of mobility was only observed with Jalisco and Nuevo León compared to Baja California (Table 4).

**Table 3:** Results from mixed-effects Poisson regression models to evaluate the influence of mobility on the incidence of positive COVID-19 cases and COVID-19 mortality rates in all states being assessed.

**Model**	**Parameter**	**IRR**	**95%CI**	***p*-value**
COVID-19 incidence *R*^2^ = 0.085	Mobility	1.052	1.048–1.055	<0.001
COVID-19 incidence adjusted *R*^2^ = 0.166	Mobility	1.052	1.048–1.056	<0.001
	Physicians per 10,000 inhabitants	1.329	1.310–1.348	<0.001
	Hospital beds per 10,000 inhabitants	0.830	0.807–0.854	<0.001
	Population living in poverty (%)	0.888	0.882–0.893	<0.001
COVID-19 mortality *R*^2^ = 0.038	Mobility	1.028	1.019–1.038	<0.001
COVID-19 mortality adjusted *R*^2^ = 0.163	Mobility	1.029	1.020–1.038	<0.001
	Physicians per 10,000 inhabitants	2.390	2.283–2.503	<0.001
	Hospital beds per 10,000 inhabitants	0.273	0.250–0.297	<0.001
	Population living in poverty (%)	1.035	1.017–1.053	<0.001

**Table 4:** Results from mixed-effects Poisson regression models to evaluate the influence of mobility on the incidence of positive COVID-19 cases and COVID-19 mortality rates per state using an interaction effect and using as reference the state of Baja California.

**Model**	**Parameter**	**IRR**	**95%CI**	***p*-value**
COVID-19 incidence *R*^2^ = 0.232	Mobility	1.016	1.012–1.020	<0.001
	Mexico City	4.406	3.715–5.225	<0.001
	Jalisco	7.815	6.717–9.092	<0.001
	Nuevo León	0.888	0.882–0.893	<0.001
	Mobility*Mexico City	1.010	1.006–1.013	<0.001
	Mobility*Jalisco	1.102	1.097–1.107	<0.001
	Mobility*Nuevo Leon	1.112	1.107–1.116	<0.001
COVID-19 mortality *R*^2^ = 0.225	Mobility	1.007	0.998–1.016	0.1233
	Mexico City	1.152	0.760–1.747	0.504
	Jalisco	13.454	9.271–19.524	<0.001
	Nuevo León	12.600	8.233–19.283	<0.001
	Mobility*Mexico City	0.999	0.991–1.007	0.726
	Mobility*Jalisco	1.130	1.117–1.142	<0.001
	Mobility*Nuevo Leon	1.115	1.102–1.128	<0.001

## Discussion

The challenge presented by the pandemic has required much more than treating a novel disease; it has demanded a social, economic, and political coordinated response (44) with many complexities deep-rooted in federated states, such as Mexico (45). NPIs, which aimed to slow viral transmission across societies, reduced population mobility globally (46). Nonetheless, NPIs implementation has required substantial changes in human behavior, resulting in heterogenous and mixed responses across different populations (15, 16, 45, 46).

Our study focuses on mobility and its correlation with morbidity and mortality in four states in Mexico. We found a significant association between mobility and incident COVID-19 cases and mortality rates using mixed-effects Poisson models. We identified that the contribution of mobility was lower for mortality (3.8%) than for incident cases (8.5%). Increasingly studies have shown that social determinants of health such as sociodemographic inequalities, differential health system capacities for critically ill patients across multiple health systems, general practitioners and nurses' ratio per inhabitant, knowledge, attitudes, and practices toward public health recommendations, in addition to the prevalence of chronic diseases impact on COVID-19 incidence and mortality rates (17, 47–54). Therefore, we adjusted all models for the number of physicians and available hospital beds per 10,000 inhabitants to proxy the impact of the availability and access to medical services in the evaluated states and the percentage of the population living in poverty. We identified that the contribution of mobility to positive COVID-19 incidence and COVID-19 mortality was sustained when adjusting for medical services and poverty. When we compared individual states with Baja California, we identified that increased mobility conferred an increased risk of incident positive COVID-19 cases in Mexico City, Jalisco, and Nuevo León. However, for COVID-19 mortality, a differential impact of mobility was only observed with Jalisco and Nuevo León compared to Baja California. This demonstrates that mobility had heterogeneous impacts on COVID-19 rates in different regions of Mexico, indicating that sociodemographic characteristics and regional-level pandemic dynamics modified the impact of reductions in mobility during the COVID-19 pandemic.

We also analyzed the correlation between mobility and COVID-19 incidence and mortality rates between the change points. This allowed us to demonstrate heterogeneity over time and across states. Findings suggest a similar pandemic course in two states, Jalisco and Nuevo Leon. In contrast, Baja California and Mexico City displayed a different trajectory.

While Mexico City and Baja California reduced their mobility by more than 40%, an upward trend of incidence and mortality rates was already in progress by the time mobility reached those levels, suggesting a late onset of NPIs in those two states. On average, incidence and mortality rates were at least 1.7 and 4.5 times higher in Mexico City and Baja California than in Jalisco and Nuevo Leon during the analysis period. Mobility analysis in Europe showed that countries with a delayed response had an 82% higher mortality rate and were forced to adopt a stricter lockdown, except in one case. Overall, countries had a difference of 11.4 days between 100 cases and their first change-point in mobility and more than 0.02 deaths per 100,000 inhabitants (18). Weaker correlations in the last weeks of the study period across the four states also suggest that decoupling between mobility and transmission occurred in the last weeks of the study period, consistent with the analysis made on numerous countries that documented a gradual decline of the relationship between mobility and transmission after strict control measure were eased in the initial stages of the pandemic, in which no data was reported on Mexico (55). Furthermore, early action allowed some countries to operate at a higher level of mobility during lockdowns without sacrificing public health. Results from published studies have shown that the effectiveness of lock-down measures, including the closure of businesses and schools, for COVID-19 containment depended largely on timely implementation and a clear distinction needs to be made when addressing this issue as physical distancing may be used interchangeably with lockdowns in other studies (19, 20).

As for mortality and mobility, correlations were positive in two of four states. Research from other countries on these two variables has yielded mixed results (18, 19, 21, 22); in this study, a significant correlation between decreased mobility and deaths was found for Jalisco and Nuevo Leon but not for Baja California and Mexico City. Positive correlations found in our study match with reports of excess mortality and mobility, which is a more objective and comparable way to assess the scale of the pandemic (56) and quantify the effects of mobility on COVID-19 cases (23).

Other authors have studied population density, which has been a predictor in the trajectory of SARS-CoV-2 epidemiology during community transmission (57, 58). In contrast, our data show that the least and most densely populated states (Baja California and Mexico City) sustained the highest incidence and mortality rates during the initial and later stages. It is worth noting that both Nuevo Leon and Baja California are border states with the United States of America and that Mexico City is the country's capital. Therefore, results could differ from other states not analyzed in this study, and further research is needed better to understand local epidemiology and transmission dynamics. In addition to the latter, it should be noted that both Jalisco and Nuevo Leon adopted stricter policies for physical distancing and COVID-19 containment compared to Mexico City and Baja California, as documented by Knaul et al. (59). This corresponds with the observed difference between these states and their respective mobility trajectory and COVID-19 epidemiology over the analyzed period in this study.

Furthermore, the adoption of staying-at-home varied through geographical regions: the northeastern border region (Nuevo Leon) had the highest adoption of this measure, at 50.1%, while the pacific-center (Jalisco) had the lowest levels, 30.6%; Mexico City and the northern pacific region (Baja California) reported adoption of 41.2 and 36.5%, respectively (60, 61). According to ENSANUT 2020, the main reason for leaving their house was buying food (70.6%), work (31.4%), buying medicines (12.1%), and going to medical consults (10%), but regarding knowledge and adoption of mitigation strategies, only 36.4% of responders identified staying-at-home as a preventive measure, while 38.5% adopted it (60).

In México, during the period analyzed in this study, controversy continuously surfaced on how the pandemic was managed by national authorities, which held leadership through the first months, and was later transferred to state authorities (12, 24, 61–65). From a perspective of public policy implementation, and according to Knaul et al. (59), “Nuevo Leon and Jalisco and its metropolitan areas of Monterrey and Guadalajara, respectively, stood out as positive examples.” According to Knaul et al., both state governments suspended non-essential activities earlier, established policies to promote social distancing before national measures were enacted, and expanded testing capacity (12). Our findings support this observation since incidence and mortality rates were lower when NPIs were implemented in Nuevo Leon and Jalisco.

The main limitation was mobility data availability since it is limited to the possibility of tracking users. Although mobile users across the states are similar (91–94.4%) (27), data may not represent all of the population, and we are aware that it might exclude some groups. Incidence data is also limited since it depends on testing strategies (12). However, correlation with mortality data validates our results. Even though multiple studies examine the relationship between NPIs and the pandemic trajectory, the most consistent and analyzed variable as a predictor among other works was population mobility (15, 16, 18–26, 55, 56). Also, we excluded the analysis of specific public policies, as all promoted NPIs in the early stages of the pandemic by national health authorities had an objective to slow viral transmission by reducing population mobility (6, 10, 59), which can be outlined as the mobility index, thus allowing for a detailed statistical analysis of the end-product of NPIs as one variable. Finally, another aspect revolving around population mobility is mobility between states, within states, cities, and sub-city areas, which is not included in our study and has shown to be associated with SARS-CoV-2 epidemiology (25, 26).

## Conclusions

NPIs focused on physical distancing were promoted during the local transmission phase, although the epidemiological trajectory varied between states. After the initial decline in mobility experienced in early April, a sustained increase in mobility followed during the rest of the country-wide suspension of non-essential activities and the return to other activities throughout mid-April and May. We identified that a 1% increase in mobility yielded a 5.2 and a 2.9% increase in the risk of COVID-19 incidence and mortality, respectively, in all evaluated states. Mobility was estimated to contribute 8.5 and 3.8% to the variability in incidence and mortality, respectively. When adjusting for medical care and poverty, the contribution of mobility to positive COVID-19 incidence and mortality was sustained. When assessing the impact of mobility in each state compared to the state of Baja California, we identified that increased mobility conferred an increased risk of incident positive COVID-19 cases in Mexico City, Jalisco, and Nuevo León. However, for COVID-19 mortality, a differential impact of mobility was only observed with Jalisco and Nuevo León compared to Baja California. We hypothesize that a contributing factor to the trajectory of the pandemic in Mexico, as occurred in other countries, was the timeliness of implementation of such measures. Our results provide valuable information for future pandemic preparedness and response against possible emerging and re-emerging pathogens of similar nature. Overall, mobility increased during the NPSD as COVID-19 cases and deaths escalated. Finally, the match between important festivities with changes in mobility could be another factor that drove mobility throughout the NPSD. Despite the continued promotion of NPIs, return to non-essential activities was encouraged by health authorities during a rising wave of cases and deaths due to political and economic pressures. Further research focused on other states and variables and governance is needed to understand the pandemic across the country thoroughly.

## Data availability statement

Publicly available datasets were analyzed in this study. This data can be found here: https://github.com/Caml1034/COVID19Mobility.git.

## Author contributions

CM-L, MC-C, and LG-G participated in the study conception and design. CM-L and MC-C acquired data. CM-L, MC-C, GD-S, EF-G, LF-R, SC-Q, NT-V, NM-R, MJ-C, KB, OB-C, and LG-G analyzed and interpreted data. CM-L, MC-C, OB-C, and LG-G contributed to the writing of the manuscript. GD-S, EF-G, LF-R, SC-Q, NT-V, NM-R, MJ-C, and KB critically revised the manuscript for important intellectual content. All authors reviewed the manuscript and granted their final approval to publish this version.

## Funding

CM-L received a scholarship from the Mexican Consejo Nacional de Ciencia y Tecnología (CVU 1012941).

## Conflict of interest

The authors declare that the research was conducted in the absence of any commercial or financial relationships that could be construed as a potential conflict of interest.

## Publisher's note

All claims expressed in this article are solely those of the authors and do not necessarily represent those of their affiliated organizations, or those of the publisher, the editors and the reviewers. Any product that may be evaluated in this article, or claim that may be made by its manufacturer, is not guaranteed or endorsed by the publisher.
