# Supplementary material for: Evaluating the impact of mobility in COVID-19 incidence and mortality: A case study from four states of Mexico
Source: Front Public Health. 2022 Aug 4;10:877800. doi: 10.3389/fpubh.2022.877800 (PMC9387383; doi:10.3389/fpubh.2022.877800)
Supplement: Supplementary file 1 [file Data_Sheet_1.docx]

Supplementary Material

## Supplementary Figures


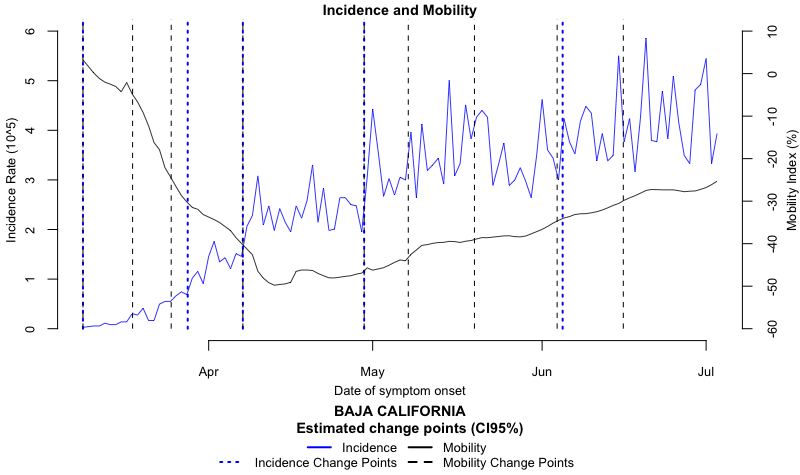


**Supplementary Figure 1.** Change points in Incidence and Mobility in Baja California, Mexico. The left vertical axis shows the incidence rate scale, 1 through 6 to the 10^5; for confirmed cases, the right vertical axis shows the mobility index in a 10% to -60% range, based on Google mobility reports. The continuous black line illustrates the mobility index; the colored continuous line shows daily confirmed cases. Change-points in mobility are shown with black vertical dotted lines and colored vertical dotted lines for the daily incidence curve.


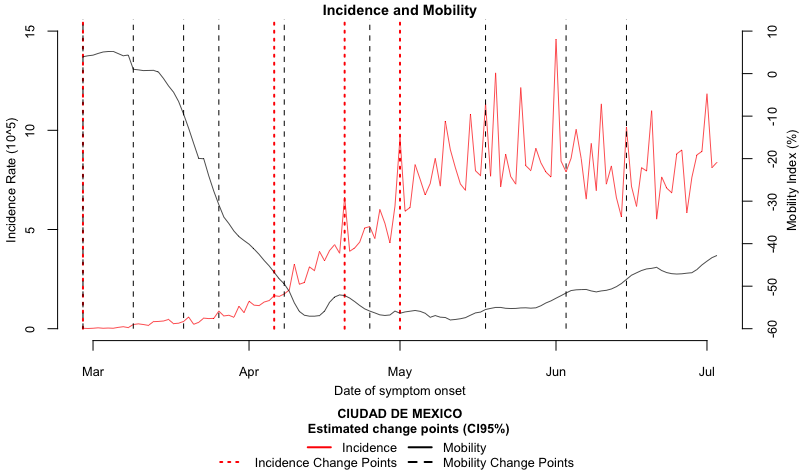


**Supplementary Figure 2.** Change points in Incidence and Mobility in Ciudad de Mexico, Mexico. The left vertical axis shows the incidence rate scale, 1 through 6 to the 10^5; for confirmed cases, the right vertical axis shows the mobility index in a 10% to -60% range, based on Google mobility reports. The continuous black line illustrates the mobility index; the continuous red line shows daily confirmed cases. Change-points in mobility are shown with black vertical dotted lines and red vertical dotted lines for the daily incidence curve.


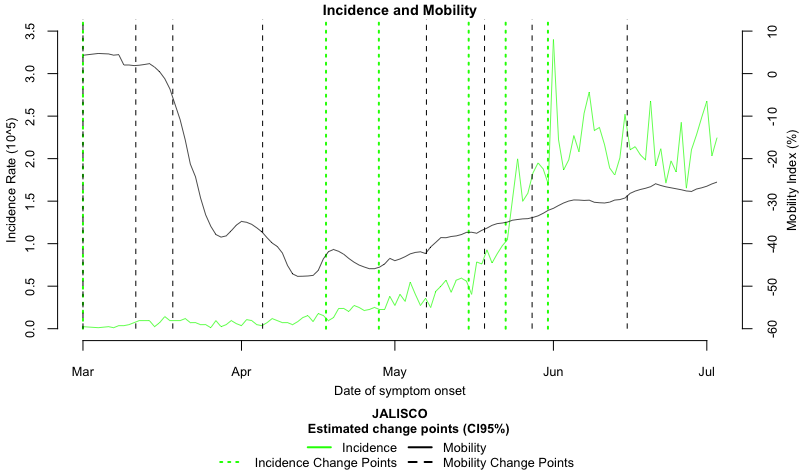


**Supplementary Figure 3.** Change points in Incidence and Mobility in Jalisco, Mexico. The left vertical axis shows the incidence rate scale, 1 through 6 to the 10^5; for confirmed cases, the right vertical axis shows the mobility index in a 10% to -60% range, based on Google mobility reports. The continuous black line illustrates the mobility index; the continuous green line shows daily confirmed cases. Change-points in mobility are shown with black vertical dotted lines and green vertical dotted lines for the daily incidence curve.


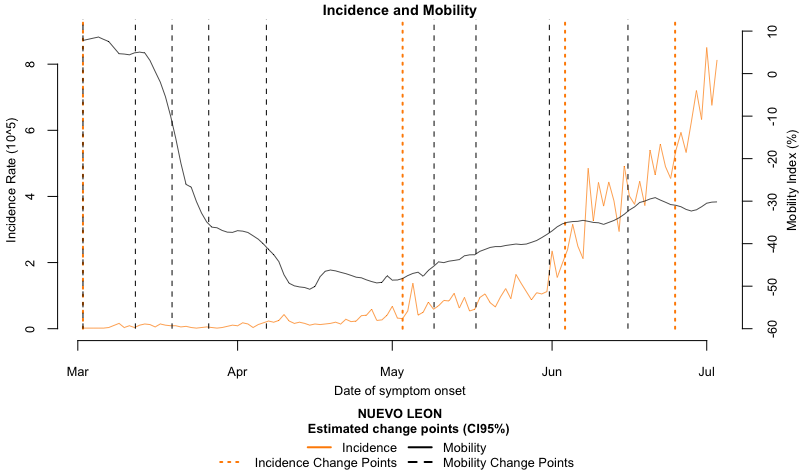


**Supplementary Figure 4.** Change points in Incidence and Mobility in Nuevo Leon, Mexico. Based on Google mobility reports, the left vertical axis shows the incidence rate, 0-8 to the 10^5 for confirmed cases, and the right vertical axis shows the mobility index in a 10% to -60% range. The continuous black line illustrates the mobility index; the continuous yellow line indicates daily confirmed cases. Change-points in mobility are shown with black vertical dotted lines and yellow vertical dotted lines for the daily incidence curve.


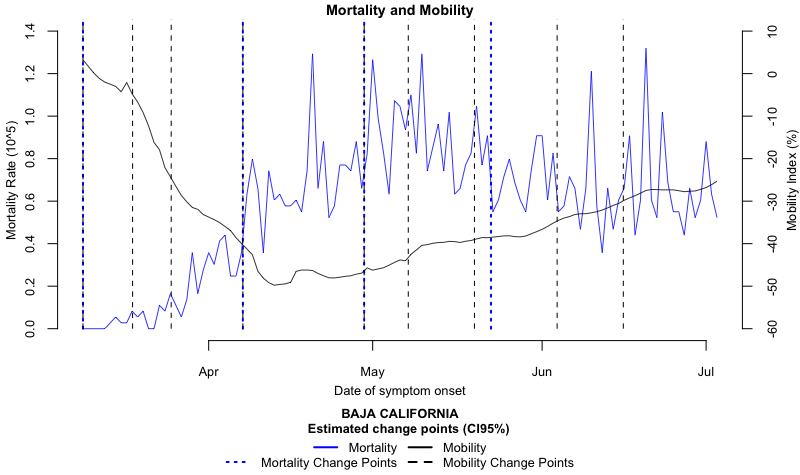


**Supplementary Figure 5.** Change points in Mortality and Mobility in Baja California, Mexico. The left vertical axis shows the mortality rate scale, 0 through 1.4 confirmed COVID-19 deaths per 100,000 habitants. Based on Google mobility reports, the right vertical axis shows the mobility index in a 10% to -60% range. The continuous black line illustrates the mobility index; the continuous blue line shows daily confirmed cases. Change-points in mobility are signaled with black vertical dotted lines and in blue vertical dotted lines for the daily deaths curve.


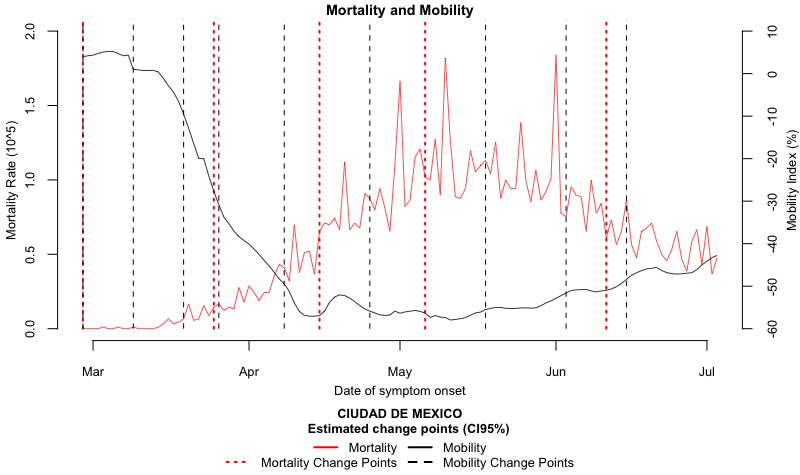


**Supplementary Figure 6.** Change points in Mortality and Mobility in Ciudad de Mexico, Mexico. The left vertical axis shows the mortality rate scale, 0 through 1.4 confirmed COVID-19 deaths per 100,000 habitants. Based on Google mobility reports, the right vertical axis shows the mobility index in a 10% to -60% range. The continuous black line illustrates the mobility index; the continuous red line shows daily confirmed cases. Change-points in mobility are signaled with black vertical dotted lines and in red vertical dotted lines for the daily deaths curve.


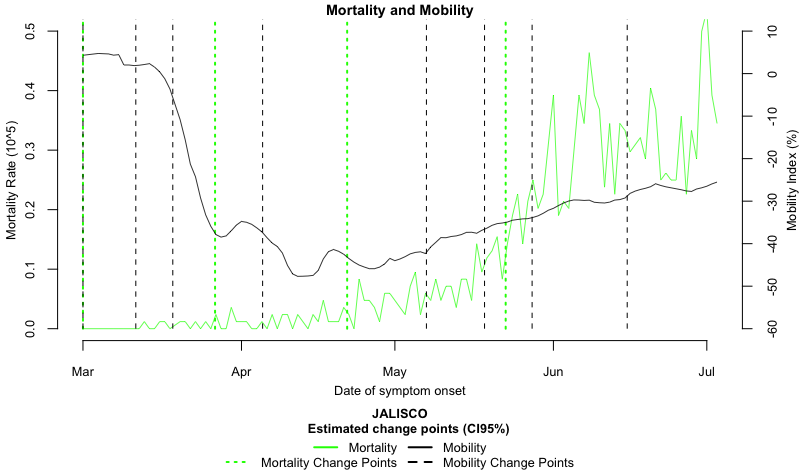


**Supplementary Figure 7.** Change points in Mortality and Mobility in Jalisco, Mexico. The left vertical axis shows the mortality rate scale, 0 through 1.4 confirmed COVID-19 deaths per 100,000 habitants. The right vertical axis shows the mobility index in a 10% to -60% range, based on Google mobility reports. The continuous black line illustrates the mobility index; the continuous green line shows daily confirmed cases. Change-points in mobility are signaled with black vertical dotted lines and in green vertical dotted lines for the daily deaths curve.


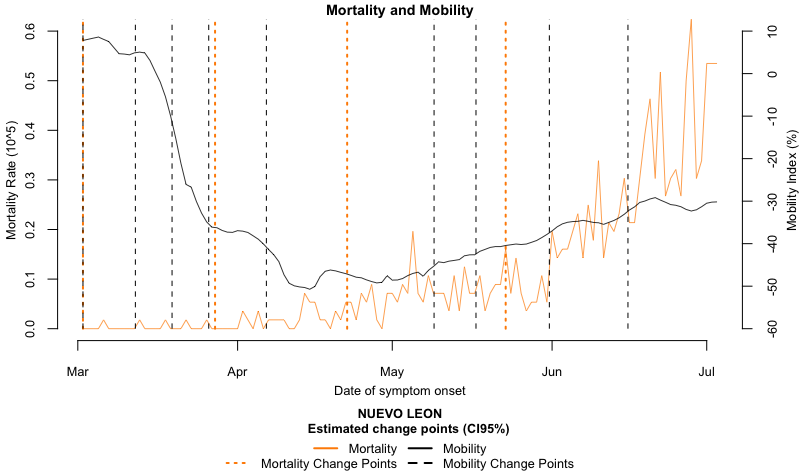


**Supplementary Figure 8.** Change points in Mortality and Mobility in Nuevo Leon, Mexico. The left vertical axis shows the mortality rate scale, 0 through 1.4 confirmed COVID-19 deaths per 100,000 habitants. Based on Google mobility reports, the right vertical axis shows the mobility index in a 10% to -60% range. The continuous black line illustrates the mobility index; the continuous yellow line indicates daily confirmed cases. Change-points in mobility are signaled with black vertical dotted lines and in yellow vertical dotted lines for the daily deaths curve.


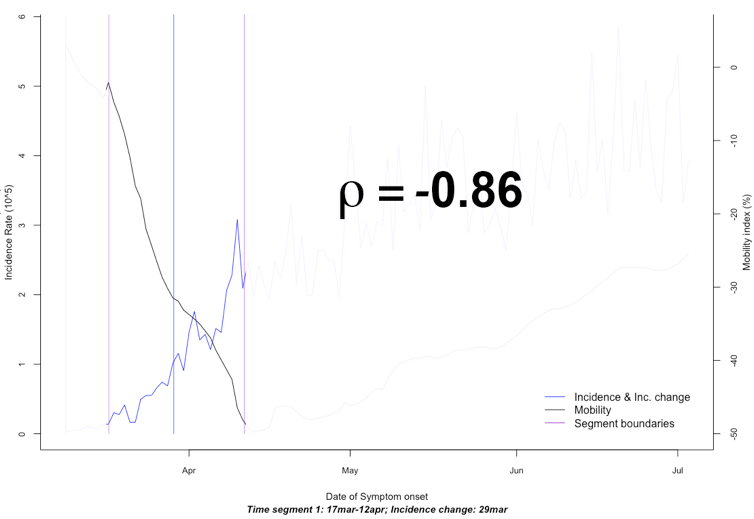

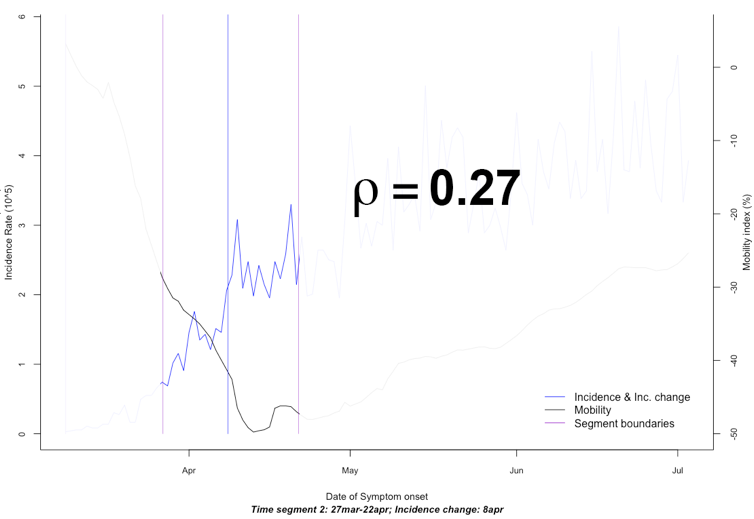

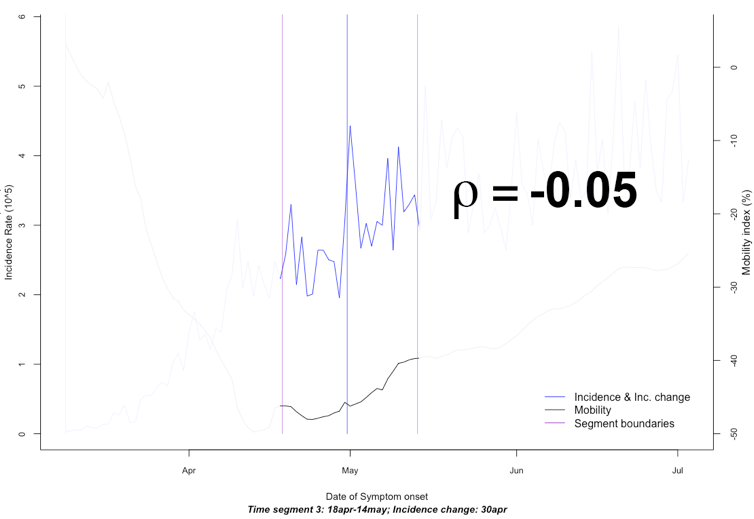

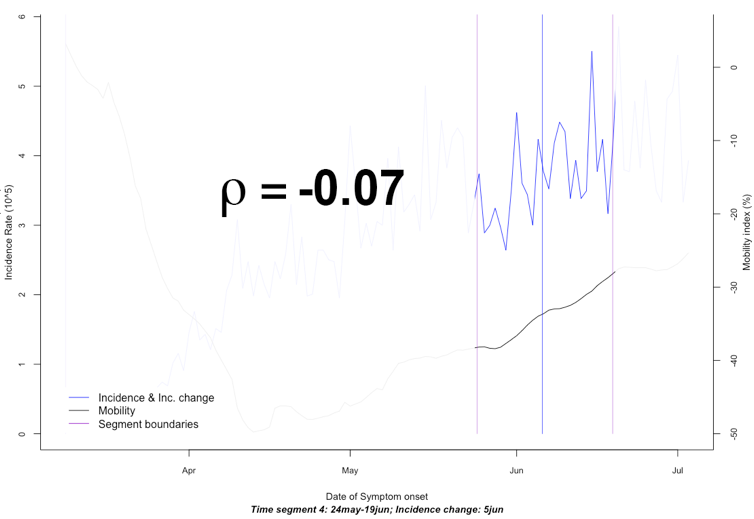


**Supplementary Figure 9.** Spearman’s Correlation Coefficient for daily incidence rate and mobility by time segments in Baja California. The left vertical axis shows the COVID-19 incidence rate per 100,000 habitants. The right vertical axis shows the mobility index in the 0% to -50% range. Time segments are delimited with purple lines. These were constructed using the change-point in incidence (vertical blue line); 12 days before and 14 days after the change-point in the incidence rate. The continuous blue line is the daily incidence rate of COVID-19 cases. Spearman’s rank-order correlation coefficients for mobility and incidence rate were calculated for each segment.

**
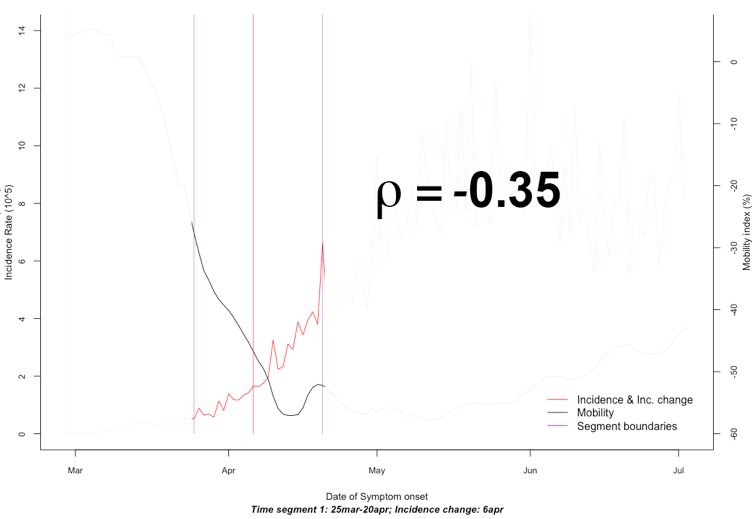

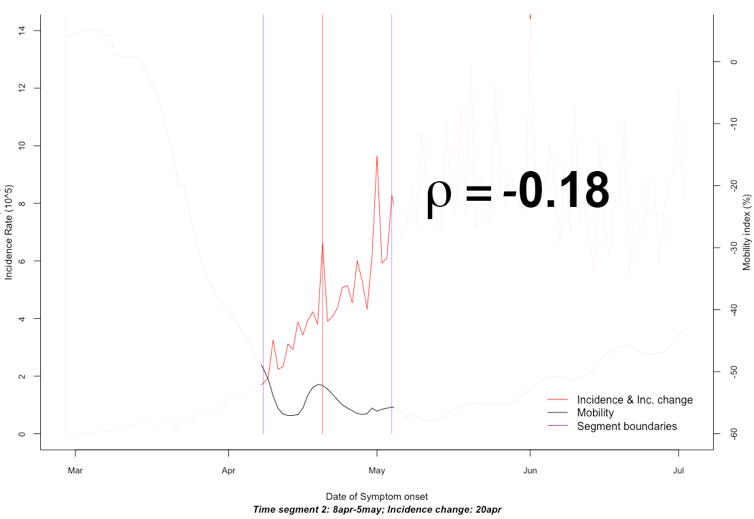

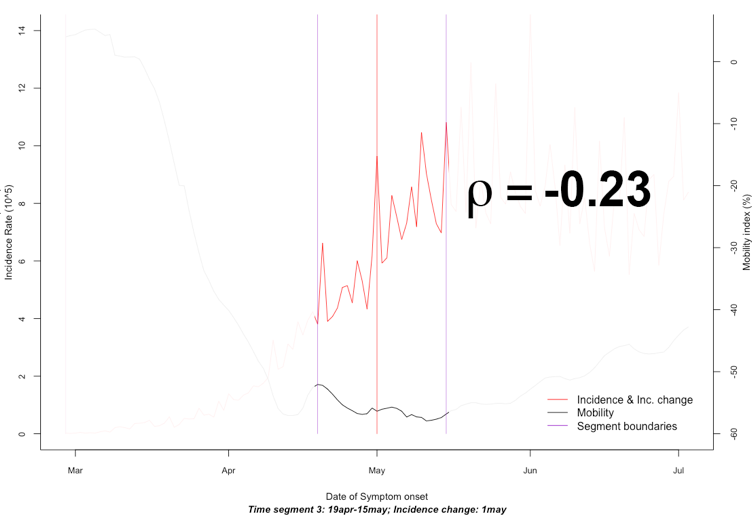
**

**Supplementary Figure 10.** Spearman’s Correlation Coefficient for daily incidence rate and mobility by time segments in Mexico City. The left vertical axis shows the COVID-19 incidence rate per 100,000 habitants. The right vertical axis shows the mobility index in the 0% to -60% range. Time segments are delimited with purple lines. These were constructed using the change-point in incidence (vertical red line); 12 days before and 14 days after the change-point. The continuous red line is the daily incidence rate of COVID-19 cases. Spearman’s rank-order correlation coefficients for mobility and incidence rate were calculated for each segment.


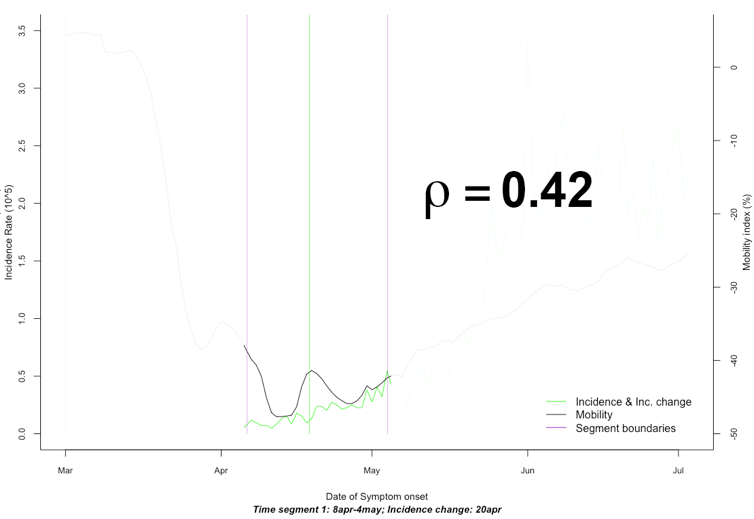

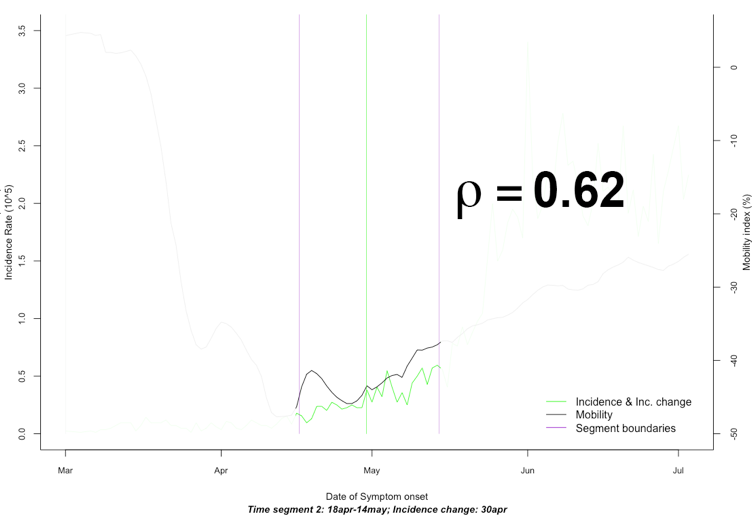

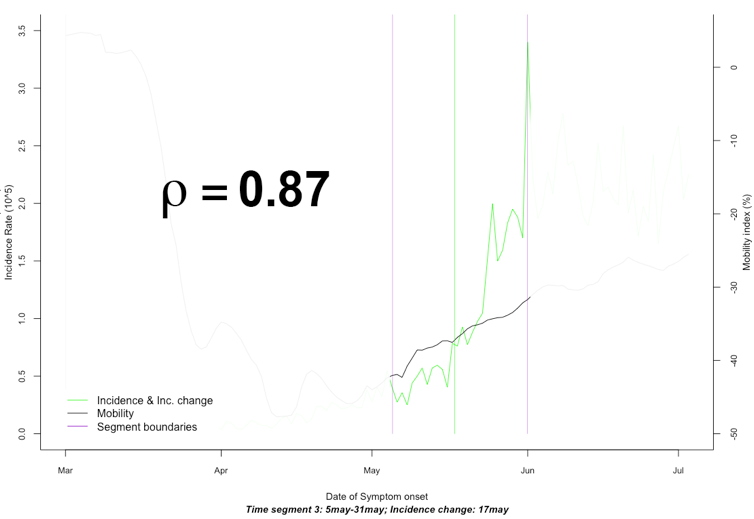

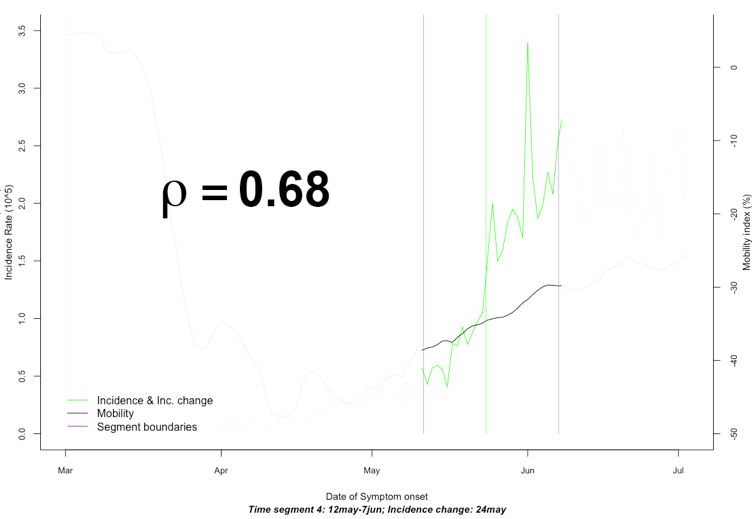

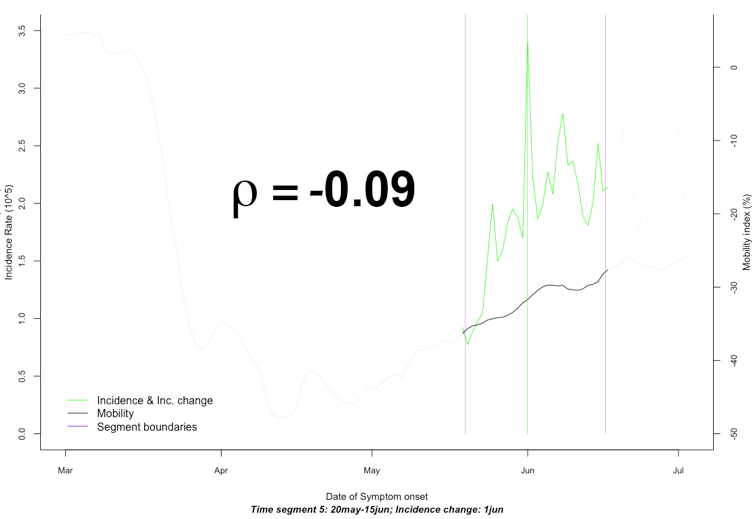


**Supplementary Figure 11.** Spearman’s Correlation Coefficient for daily incidence rate and mobility by time segments in Jalisco. The left vertical axis shows the COVID-19 incidence rate per 100,000 habitants. The right vertical axis shows the mobility index in the 0% in the -50% range. Time segments are delimited with purple lines. These were constructed using the change-point in incidence (vertical green line); 12 days before and 14 days after the change-point in the incidence rate. The continuous black line represents the daily mobility index. The continuous green line is the daily incidence rate of COVID-19 cases. Spearman’s rank-order correlation coefficients for mobility and incidence rate were calculated for each segment.


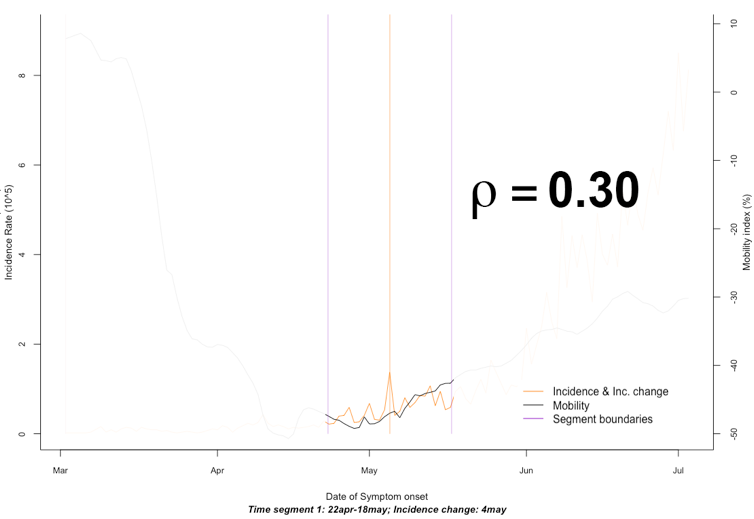

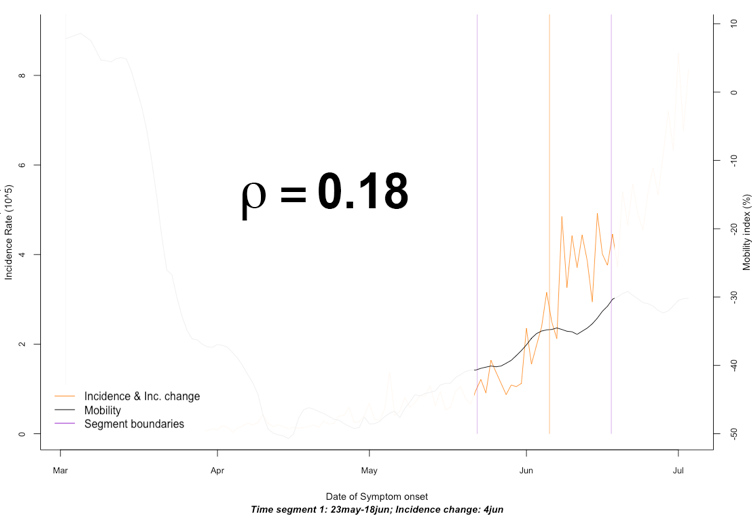

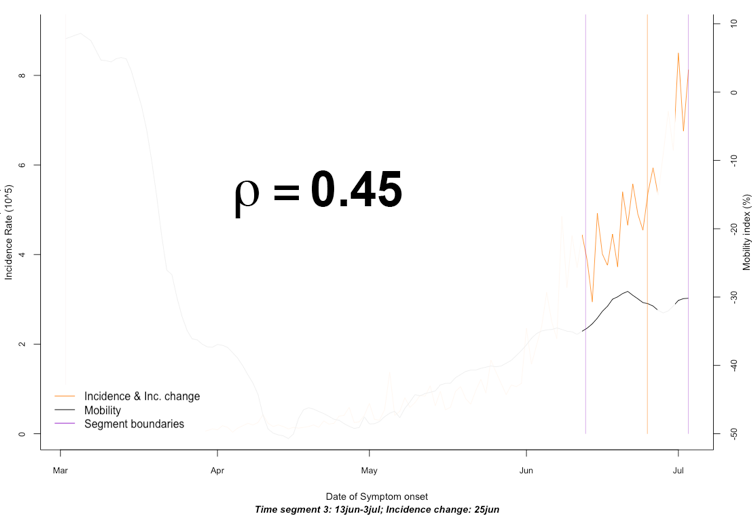


**Supplementary Figure 12.** Spearman’s Correlation Coefficient for daily incidence rate and mobility by time segments in Nuevo León. The left vertical axis shows the COVID-19 incidence rate per 100,000 habitants. The right vertical axis shows the mobility index in the 0% to -50% range. Time segments are delimited with purple lines. These were constructed using the change-point in incidence (vertical blue line); 12 days before and 14 days after the change-point in the incidence rate. The continuous black line represents the daily mobility index. The continuous blue line is the daily incidence rate of COVID-19 cases. Spearman’s rank-order correlation coefficients for mobility and incidence rate were calculated for each segment.


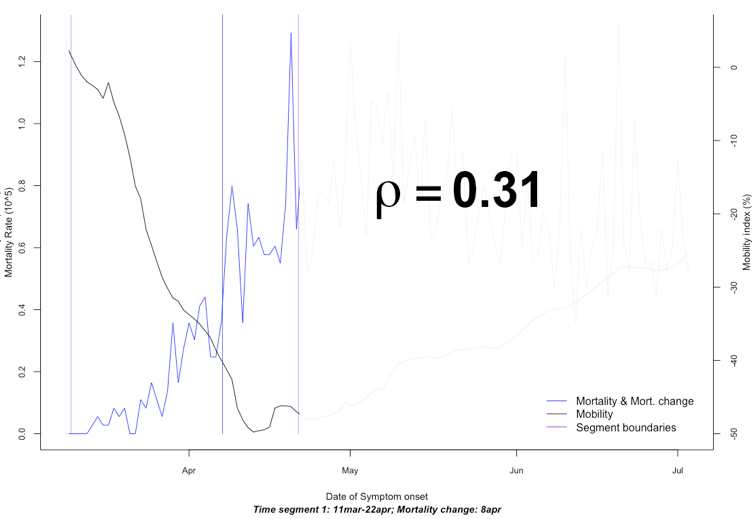

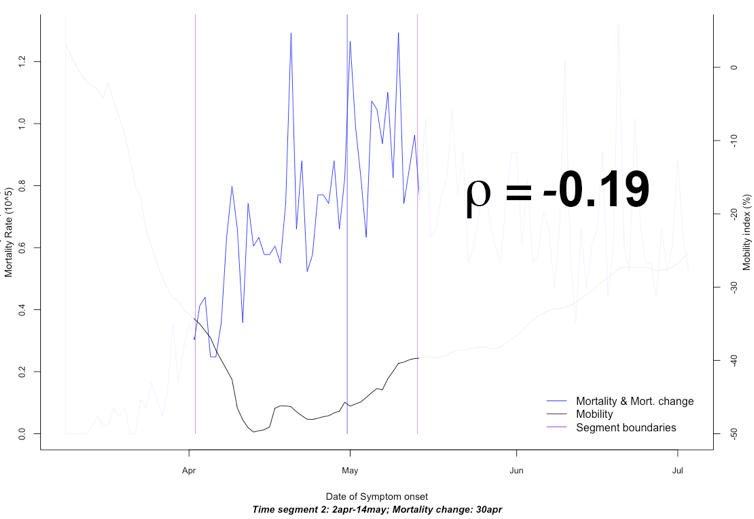

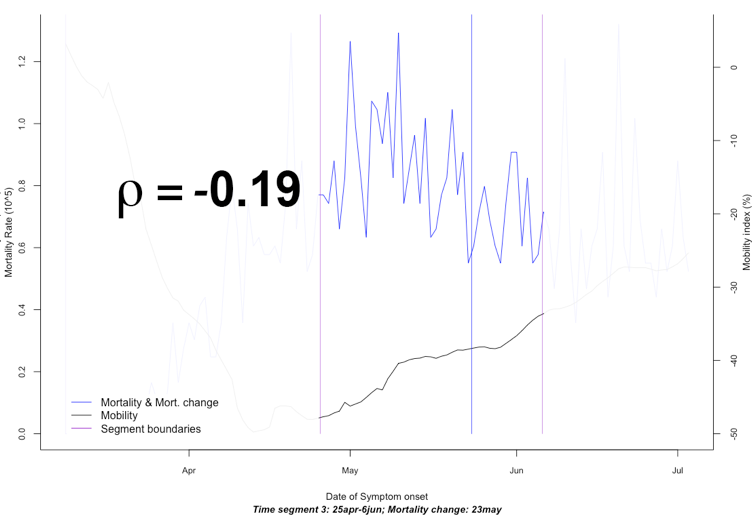


**Supplementary Figure 13.** Spearman’s Correlation Coefficient for daily mortality rate and mobility by time segments in Baja California. The left vertical axis shows the COVID-19 mortality rate per 100,000 habitants. The right vertical axis shows the mobility index in the 0% to -50% range. Time segments are delimited with purple lines. These were constructed using the change-point in mortality (vertical blue line), 28 days before and 14 days after the change-point in the mortality rate. The continuous black line represents the daily mobility index. The continuous blue line is the daily mortality rate of COVID-19 cases. Spearman’s rank-order correlation coefficients for mobility and mortality rate were calculated for each segment.

**
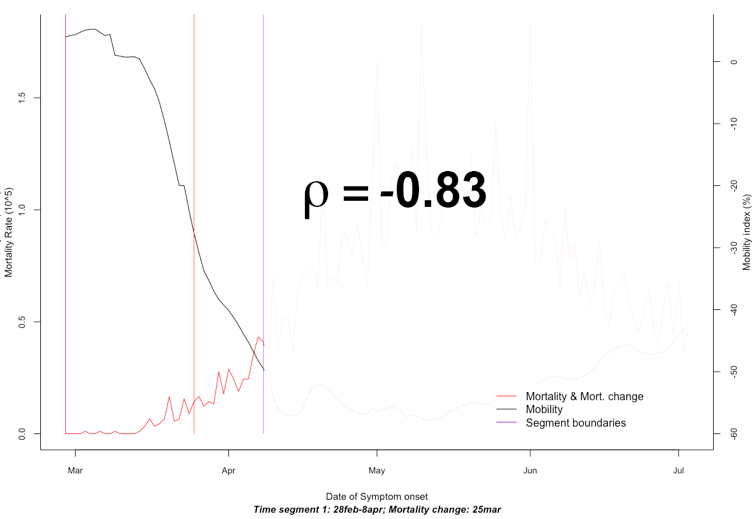

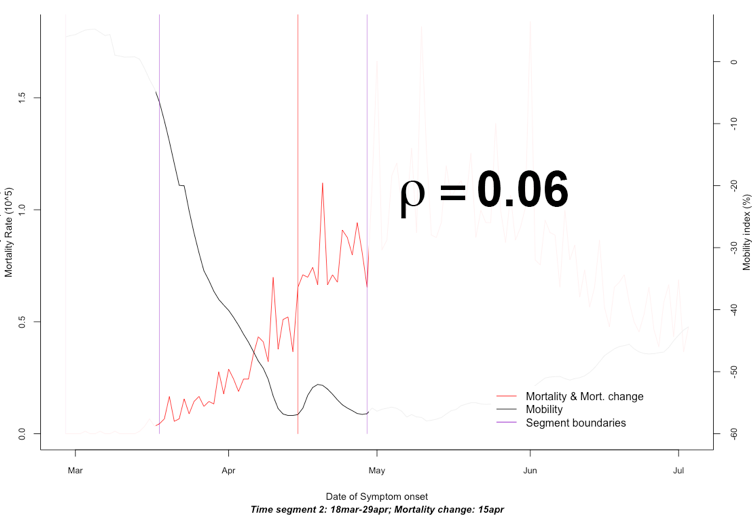

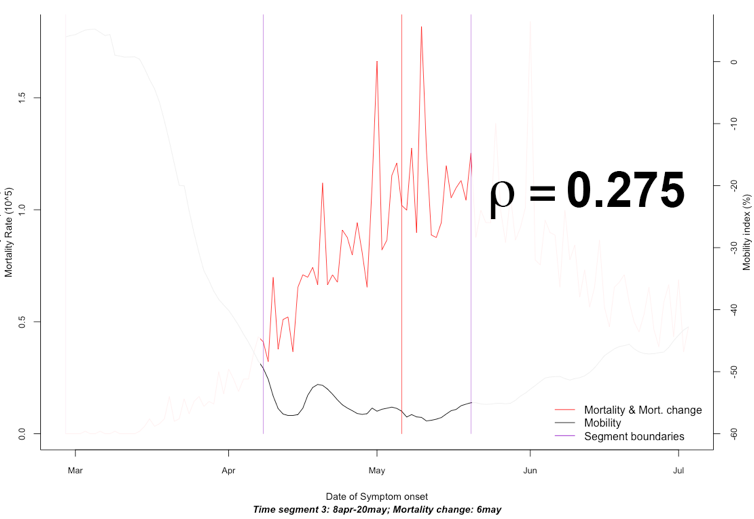

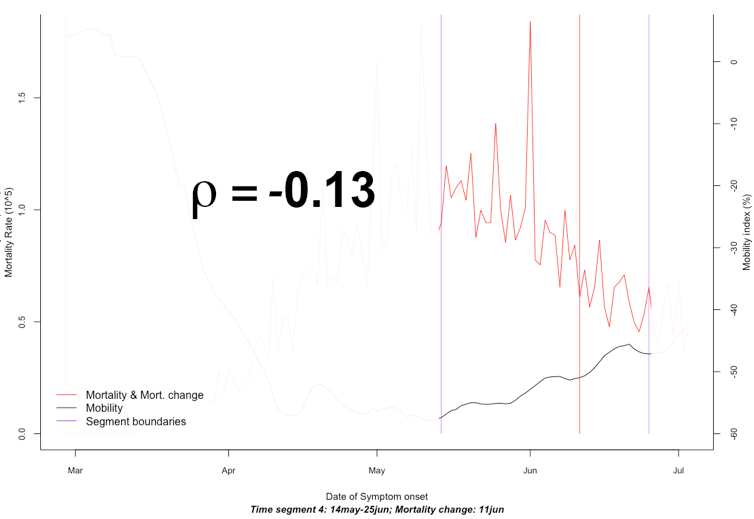
**

**Supplementary Figure 14.** Spearman’s Correlation Coefficient for daily mortality rate and mobility by time segments in Mexico City. The left vertical axis shows the COVID-19 mortality rate per 100,000 habitants. The right vertical axis shows the mobility index in the 0% to -60% range. Time segments are delimited with purple lines. These were constructed using the change-point in mortality (vertical red line); 28 days before and 14 days after the change-point. The continuous black line represents the daily mobility index. The continuous red line is the daily mortality rate of COVID-19 cases. Spearman’s rank-order correlation coefficients for mobility and mortality rate were calculated for each segment.

**
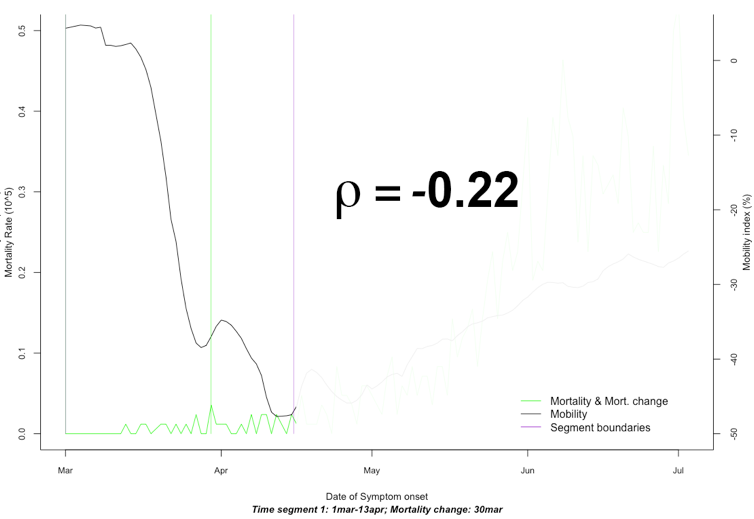

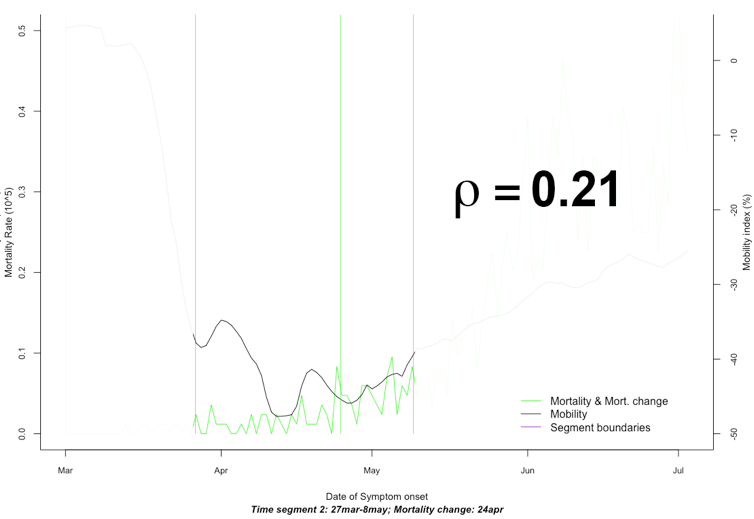

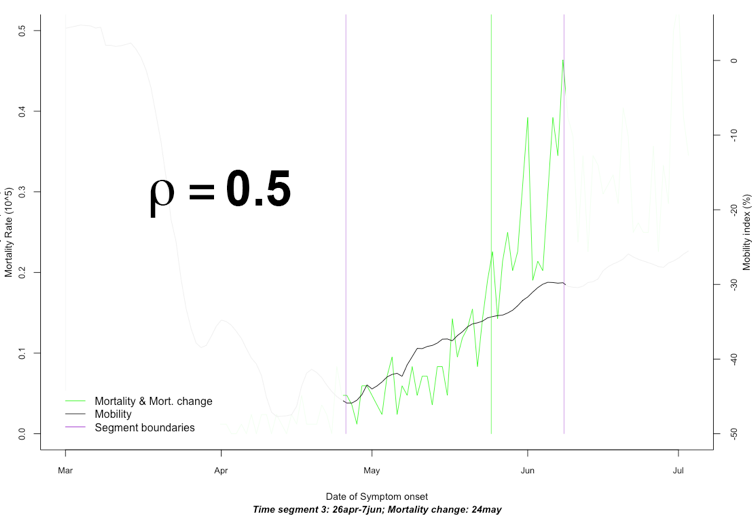
**

**Supplementary Figure 15.** Spearman’s Correlation Coefficient for daily mortality rate and mobility by time segments in Jalisco. The left vertical axis shows the COVID-19 mortality rate per 100,000 habitants. The right vertical axis shows the mobility index in the 0% in the -50% range. Time segments are delimited with purple lines. These were constructed using the change-point in mortality (vertical green line), 28 days before and 14 days after the change-point in the mortality rate. The continuous black line represents the daily mobility index. The continuous blue line is the daily mortality rate of COVID-19 cases. Spearman’s rank-order correlation coefficients for mobility and mortality rate were calculated for each segment.

**
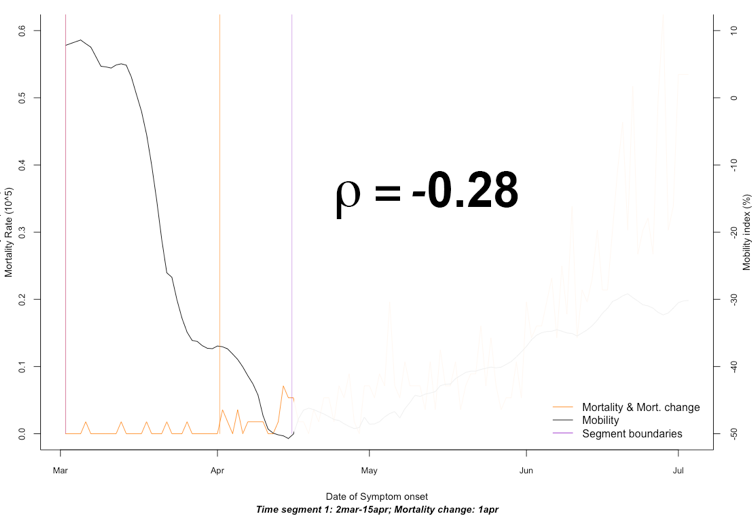

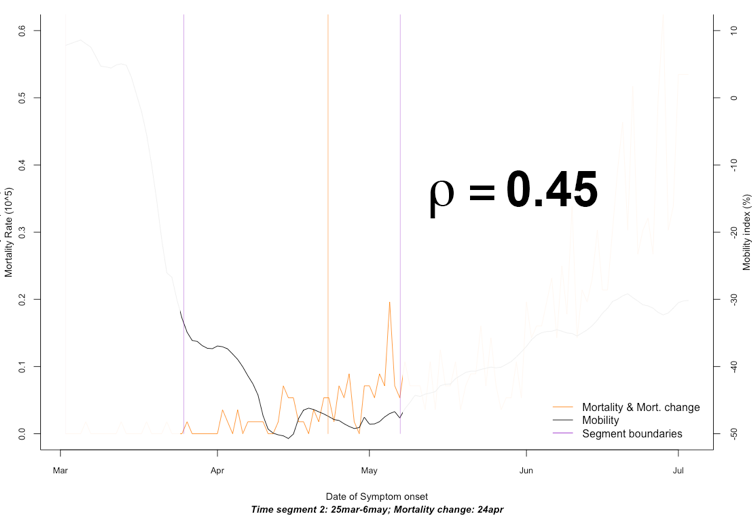

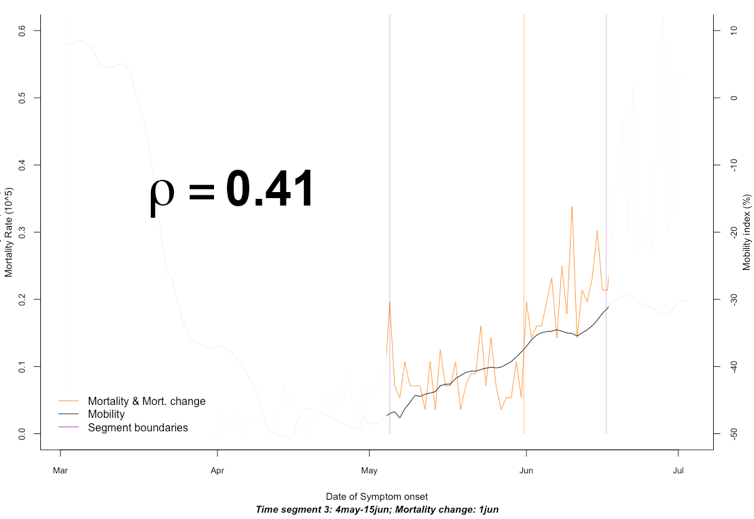
**

**Supplementary Figure 16.** Spearman’s Correlation Coefficient for daily mortality rate and mobility by time segments in Nuevo León. The left vertical axis shows the COVID-19 mortality rate per 100,000 habitants. The right vertical axis shows the mobility index in the 0% to -50% range. Time segments are delimited with purple lines. These were constructed using the change-point in mortality (vertical blue line), 28 days before and 14 days after the change-point in the mortality rate. The continuous black line represents the daily mobility index. The continuous blue line is the daily mortality rate of COVID-19 cases. Spearman’s rank-order correlation coefficients for mobility and mortality rate were calculated for each segment.
